# Non-specific effect of double-stranded RNAs on Egyptian broomrape (*Phelipanche aegyptiaca*) seed germination

**DOI:** 10.3389/fpls.2024.1492738

**Published:** 2025-01-14

**Authors:** Nariman Zainali, Houshang Alizadeh, Hassan Alizadeh, Philippe Delavault

**Affiliations:** ^1^ Department of Agronomy and Plant Breeding, College of Agriculture and Natural Resources, University of Tehran, Karaj, Iran; ^2^ Unité en Sciences Biologiques et Biotechnologies, UMR 6286, Nantes Université, Centre National de la Recherche Scientifique (CNRS), Nantes, France

**Keywords:** broomrape, CYP707A1, dsRNA, germination, nitrogen, nucleotides, RNAi

## Abstract

Obligate root parasitic plants of the Orobanchaceae family exhibit an intricate germination behavior. The host-dependent germination process of these parasites has prompted extensive research into effective control methods. While the effect of biomaterials such as amino acids and microRNA-encoded peptides have been explored, the effect of double-stranded RNAs (dsRNAs) has remained unexamined during the germination process. In this study, we asked whether an exogenously applied dsRNA can inhibit the germination of a root parasite, *P. aegyptiaca*. To this end, a dsRNA was designed to target the *CYP707A1* (dsCYP7), a marker gene of the chemically-dependent germination of broomrape seeds. Application of a concentrated dsCYP7 significantly reduced seed germination. However, two non-germination-specific dsRNAs designed to target mannose-6-phosphate reductase and green fluorescent protein brought about similar inhibitions. Moreover, applying rNTPs and dNTPs, which mimic nitrogenous bases of nucleic acids, also caused a similar reduction in germination, suggesting that the non-specific inhibitory effect of the dsRNAs might arise from their nucleotides. While dsRNA application inhibited seed germination, their non-specific effects may pose a challenge for their application in studying root parasites germination. This underscores the importance of finding solutions to minimize the non-specific effects of dsRNAs to improve the potential of dsRNA as a tool to study and control root parasitic plants.

## 1 Introduction

The process of seed germination in many orobanchaceous parasitic plants is captivatingly intricate. These parasites produce a huge number of minuscule seeds with long-term viability in the soil (Joel, 2013) and germinate only under specific conditions. After a conditioning period during which seeds absorb water and get imbibed, seeds do not germinate unless they receive a chemical signal, called germination-stimulant (Nelson, 2021). Most of these chemical signals belong to the strigolactones (SLs) phytohormone family (Huizinga and Bouwmeester, 2023), and are secreted by the host plants, especially in phosphorus- and nitrogen-depleted conditions (Yoneyama et al., 2007b, 2007a, 2012). The perception of SLs by KAI2 receptors in parasites breaks the seed dormancy and leads to germination, and eventually parasitism (Brun et al., 2021).

In the past few years, our overall knowledge of the molecular events that lead to germination has considerably been improved. However, given the complexity of these processes, there is an increasing need for effective molecular tools to dissect the underlying mechanisms of germination and parasitism in these plants (Yoshida and Kee, 2021). RNA interference (RNAi) has emerged as a promising approach, allowing for the targeted silencing of specific genes, offering a versatile tool not only to study but also to control seed germination in parasitic plants. While RNAi has been effective in silencing genes at post-attachment stages, its potential to influence the pre-attachment phases, such as seed germination, has still remained unexplored. A few studies have shown the functionality of dsRNAs absorbed via seed or root systems in silencing their target genes in model plant species (Jiang et al., 2014; Li et al., 2015; Ludba, 2018). Moreover, a recent new study has opened up a new avenue for environmental RNAi experiments in plants. Betti et al. (2021) showed that the exogenously applied miR399 through the growth medium triggered the silencing of its target gene, *PHO*, in *Arabidopsis thaliana* plants after being absorbed through the root system and movement via the xylem channels. In addition, culturing plants overexpressing the miR399 reduced the expression level of its target gene in the neighboring wild-type *Arabidopsis* plants likely by secreting miRNAs into the environment (Betti et al., 2021). More recently, Tourneur and colleagues (2024) showed that miRNA-encoded peptides (miPEPs) can influence the germination of *Orobanche cumana* when applied exogenously. The researchers used various synthetic miPEPs on the *O. cumana* seeds and found that specific miPEPs significantly inhibited the seed germination by increasing the pri-miRNAs expression and their corresponding target genes downregulation (Tourneur et al., 2024). These lines of evidence may suggest that the process of germination in root holoparasites can be controlled via the manipulation of the genes involved in the germination process through gene silencing (Zainali et al., 2024b).

To test this hypothesis, an experiment was conducted by exogenously applying a dsRNA to manipulate seed germination in *P. aegyptiaca*. Among several critical genes that are involved in the germination process of broomrapes (Zainali et al., 2024b), we selected the *CYP707A1*, an abscisic acid (ABA)-catabolic gene, which has been shown to be up-regulated following treatment with GR24, a synthetic strigolactone analog, and is necessary for the release of dormancy in broomrape species (Lechat et al., 2012; Brun et al., 2019).

## 2 Materials and methods

### 2.1 Plant materials

Seeds of *Phelipanche aegyptiaca* (Pers.) Pomel were collected in September 2020 from infested tomato fields in Sanandaj, Kurdistan (Iran). After sieving through 400, 250, 180, and 165 μm strainers, the fraction between 400-250 μm was collected and stored at 21°C until use.

### 2.2 Seed disinfection and conditioning

Seed disinfection was carried out according to Pouvreau et al. (2021). Seeds, in the final density of 10-20 g/L (dry seed weight/v), were added with incubation medium (1 mM HEPES buffer pH 7.5, and 0.1% plant preservative mixture (PPM, v/v)), tubes were wrapped with aluminum foil and kept in dark at 21°C for four days before (+)GR24 induction.

### 2.3 Seed and dilution plates preparation

Four days post-conditioning, the incubation media was discarded and equal volumes of dH_2_O and 0.1% sterile agarose were added to set seed density at 10-20 g/L. Afterward, HEPES and PPM were added as previously described. Finally, seeds were distributed into the 96-well plate (Cell Culture Multiwell Plate Cellstar; Greiner Bio-One) using cut DISTRITIPS^®^ (Gilson, France) in a required volume. A dilution plate was prepared for (+)GR24 containing 10^-5^ to 10^-12^ M concentrations in 1% acetonitrile.

### 2.4 Preparation of dsRNAs

#### 2.4.1 Designing dsRNAs

dsCYP7 was designed as explained in protocol 1 (Supplementary material, SM). The same procedure was used to design the dsGFP while the same sequence as Farrokhi et al. (2019) was used for dsM6PR.

#### 2.4.2 Inducing *PaCYP707A1* expression

Disinfected *P. aegyptiaca* seeds were kept at 21°C in the dark for seven days in the conditioning medium. After seven days, seeds were treated with 10^-6^ M (+)GR24 for maximal germination induction (Yao et al., 2016). Subsequently, seeds were incubated at 21°C in the dark for up to 18 hours for maximal *CYP707A1* upregulation (Lechat et al., 2012). Afterward, seeds were blotted and dried on tissue paper, transferred into aluminum foil, and immediately snap-frozen in liquid nitrogen (N_2_).

#### 2.4.3 RNA isolation and cDNA preparation

Frozen seeds were ground to a fine powder in pre-chilled mortars in liquid N_2_. RNA was isolated from 100 mg starting seed materials using the NucleoSpin-RNA-Plant kit (MACHEREY-NAGEL, Germany) as per manufacturer’s instruction. The isolated RNAs were treated with RNase-free DNase I set (QIAGENE) for effective removal of DNAs. Samples were then purified using the NucleoSpin RNA Clean-up XS kit (MACHEREY-NAGEL, Germany) and eluted in 20 µL RNase-free H_2_O. The quantity and quality of RNA samples were measured spectrophotometrically and electrophoretically. The first cDNA strand was synthesized from one μg total RNA using the qScript cDNA SuperMix (Quantabio) according to the manufacturer’s instruction.

#### 2.4.4 Amplification of the gene fragments

Two gene-specific primers were designed using Primer3 (Untergasser et al., 2012) to amplify the corresponding region of the CYP7 gene fragment (242 bp). Moreover, the T7 promoter sequence was added to the 5′ ends of each primer (**Table 1**). The corresponding fragments for dsM6PR (340 bp) and dsGFP (214 bp) in the L4440 vectors containing them were amplified using the T7 primers presented in **Table 1**. PCR amplification was carried out in a volume of 100 µL containing 100 µM dNTPs (Promega), 25 µM of each primer, 1 U *Q5* DNA polymerase (New England Biolabs), 2 µL of the first cDNA strand for CYP7, and 10 ng L4440 vectors for M6PR and GFP. Amplification was carried out in a MyCycler thermal cycler (BioRad, USA). The thermal program included an initial denaturation step at 98°C for 2 minutes, followed by 35 cycles of 10 seconds at 98°C, 30 seconds at 60°C, 30 seconds at 72°C, and ended with a final extension step at 72°C for 2 minutes. Five µL of the amplification reactions were run in the electrophoresis for 30 minutes at 100 V in 1% agarose gel. The PCR reaction was purified with the NucleoSpin Gel and PCR Clean-up kit (MACHEREY-NAGEL, Germany) according to the manufacturer’s protocol. The purified fragments were then eluted in 20 µL of RNase-free H_2_O. The sequence of the amplified *PaCYP707A1* fragment was also confirmed via Sanger sequencing.

**Table 1:** List of the primers used in the current study.

Primers	Sequence (5’ to 3’)	Length (nt)
T7.PaCYP7.F	GCG**TAATACGACTCACTATAGGG**CGCAAGCTGTCACTGAAGAGC	44
T7.PaCYP7.R	GCG**TAATACGACTCACTATAGGG**CAGGATTGTGGTGAATGTTTCTGA	47
T7.F	GCG**TAATACGACTCACTATAGGG**CGAATTG	30
T7.R	AAT**TAATACGACTCACTATAGGG**AGACCGG	30

The nucleotides in red were added randomly to avoid primer erosion during and after amplification. The black capital letters are the gene-specific part of the primers. **Bold capital** letters are the minimum T7 promoter sequence.

#### 2.4.5 *In vitro* transcription and dsRNA purification

The *in vitro* transcription reactions were performed according to the MEGAscript Kit manual (Invitrogen) in a total volume of 20 µL. The *in vitro* transcribed dsRNAs were then purified using the MEGAclear kit (Invitrogen) according to the manufacturer’s protocol and were eluted in 100 µL sterile dH_2_O. The quantity and quality of dsRNAs were measured spectrophotometrically and electrophoretically. Moreover, the picomolar concentration of the dsRNAs was calculated using the RNA Molecular Weight Calculator tool (https://www.aatbio.com/tools/calculate-RNA-molecular-weight-mw, protocol 2, SM).

#### 2.4.6 Concentrating dsRNAs

To obtain a more concentrated dsRNA, ethanol precipitation with ammonium acetate was performed according to the MEGAclear kit instruction (Invitrogen). To this end, several purified *in vitro* transcription reactions were pooled and used for precipitation. At the end, the dsRNA pellets were resuspended in a desired volume of sterile dH_2_O for seed treatment.

### 2.5 Treating broomrape seeds with dsRNAs

In the first experiment, dsCYP7 was applied to the final concentration of ~ 0.7 µM in a gradient of (+)GR24 (10^-6^-10^-13^ M). A dilution plate was prepared as previously described and the seed plate was prepared according to the layout presented in **Supplementary Figure S1**. In the second experiment, a more concentrated dsCYP7 of ~ 12 µM was applied on the seeds in the presence of 10^-6^-10^-8^ M (+)GR24 (**Supplementary Figure S2**).

However, in the subsequent experiments, three concentrations of ~ 6, 3, and 1.5 µM were applied for dsCYP7, dsM6PR, and dsGFP (**Supplementary Figures S3, S4**). In addition, ribonucleotides (rNTPs, 50 mM stock solution, Invitrogen) and deoxyribonucleotides (dNTPs, 50 mM stock solution, Promega) were used in the final concentrations of ~ 5, 2.5, and 1.25 mM (protocol 3, **Supplementary Figure S4**, SM). It is worth noting that in these experiments, 10^-6^ M (+)GR24 was applied for germination induction. After treatment, the seed plates were kept at 21°C in the dark for four days.

### 2.6 Staining and absorbance reading

In the experiment with 0.7 µM dsCYP7, seeds were added with 5 µL Thiazolyl Blue Tetrazolium Bromide (MTT, 5 g/L, SIGMA-Aldrich) per well eight days post-treatment (8-dpt) and kept at 21°C in dark for one day. The day after, 100 µL of solubilization buffer (10% Triton X-100 and 0.04 M HCl in isopropanol) was added per well.

### 2.7 Data collection and analysis

The seed plates were regularly monitored from day one to eight after treatment under a binocular (Olympus SZX10; Olympus Europa GmbH) and the number of germinated seeds was determined. The seeds with protruded radicals were considered as germinated throughout our experiments. The relative germination ratio of each well was calculated as below:


$$Relative\;germination\;rate=\;\frac{\%\;germination\;of\;the\;well-\%\;germination\;of\;blank}{\%\;germination\;of\;control^{+}-\%\;germination\;of\;blank}$$

where control+ was the germination of the wells containing 10^-6^ M (+)GR24 and blank was the germination of the wells without (+)GR24. To make the comparison of the germination rates possible, seeds treated with 10^-6^ M (+)GR24 were considered as positive control, and seeds untreated with (+)GR24 or treated with 10^-13^ M (+)GR24 were considered as negative control as the highest and lowest seed germinations were achieved in these groups, respectively.

In the experiment with 0.7 µM dsCYP7, 570 and 630 absorbances were read with a Polarstar Omega using an UV/Vis spectrometer (BMG Labtech) after MTT staining. The dose-response curve was obtained as described previously (Pouvreau et al., 2021) using the *drc* package V3.0.1 (Ritz et al., 2015) in R V4.3 (R Core Team, 2023). In this experiment, the absorbances of the wells were used to obtain the dose-response curve.

However, in the experiment with 12 µM dsCYP7, the relative germination rates were directly calculated from the germination rate of the wells and were used to obtain the dose-response curve because the wells containing dsCYP7 showed background color making absorbance reading impossible. The effect of dsCYP7 on the seed germination was statistically determined using the non-parametric Mann-Whitney U test as the relative absorbance and germination rates were not normally distributed.

In the rest of the experiments, the relative germination rates were also calculated from the germination rates of the wells. The analysis of variance (ANOVA) was carried out to infer the statistical differences among the treatments in the subsequent experiments as the calculated relative germination ratios were either normally distributed or log-normal. The analysis was followed by Tukey’s honestly significant difference test (Tukey’s HSD) for multiple pairwise comparisons.

Minitab V21.4.2 was used for statistical analyses and data were visualized using the *ggplot2* package V3.5.0 (Wickham, 2016) in R V4.3 (R Core Team, 2023).

## 3 Results

### 3.1 Amplification of the *PaCYP707A1* gene fragment and dsCYP7 preparation

The result of BLASTp for the *PaCYP707A1* sequence retrieved from the PPGP database showed the ABA 8’-hydroxylase protein in *P. ramosa* as the best hit indicating that the sequence corresponded to the same gene in *P. aegyptiaca*. Moreover, Sanger sequencing showed that the amplified *PaCYP707A1* fragment was almost identical to the sequence retrieved from the PPGP database (**Supplementary Figure S5**). The agarose gel electrophoresis confirmed the production of the corresponding products with their expected sizes of ~290 and ~240 bp for the *PaCYP707A1* PCR product and dsCYP7, respectively. Considering that the *PaCYP707A1* fragment contained two T7 promoter sequences, its size was larger than that of dsCYP7 (**Supplementary Figure S6**).

### 3.2 Exogenous application of dsRNAs on *P. aegyptiaca* seeds

#### 3.2.1 dsCYP7 treatment in a gradient of (+)GR24

**Figure 1:**
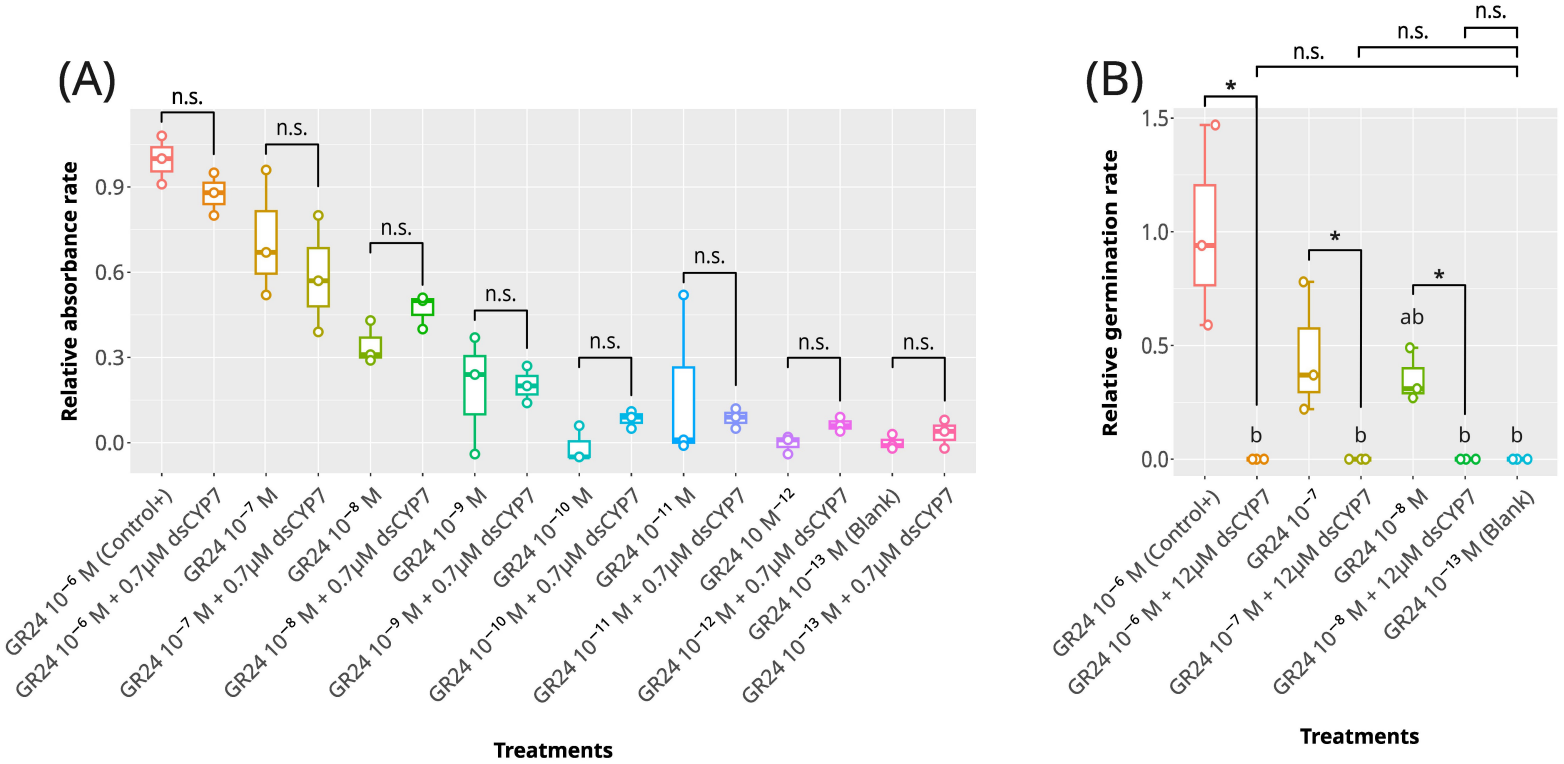
The effect of dsCYP7 treatment on the germination of Egyptian broomrape, *P. aegyptiaca*. **(A)** The boxplot for the relative absorbance rates for the experiment with 0.7 µM dsCYP7. 10^-6^ M +GR24 was considered as the positive control and seeds treated with 10^-13^ M +GR24 were considered as blank. **(B)** The boxplot for the relative germination rates for the experiment with 12 µM dsCYP7. The effect of dsCYP7 on the seeds in each +GR24 concentration (10^-6^-10^-13^ M) was statistically determined using the Mann-Whitney U test. Pairs marked with asterisk are significantly different at *p-value* = 0.05. n.s, non-significant.

Initially, dsCYP7 was applied to the seeds at the final concentration of 0.7 µM in a gradient of (+)GR24 (**Supplementary Figure S1**). The seed plate was monitored regularly from day one to eight after treatment. Staining with MTT and germination bioassays were conducted to check the seed viability state 8-dpt. No significant changes in the relative absorbances were observed in the seed treated with 0.7 µM dsCYP7 compared with seeds treated only with (+)GR24 up to 8-dpt (*p-value* > 0.05, **Figure 1A**; **Supplementary Figure S7**) indicating that dsCYP7 had no or negligible impact on the seed germination at the applied concentration.

Therefore, a more concentrated dsCYP7 at the final concentration of ~ 12 µM was applied on the seeds in the presence of 10^-6^-10^-8^ M (+)GR24 (**Supplementary Figure S2**). No radical protrusion, seed germination, or seedling growth was observed in the wells with 12 µM dsCYP7 up to 7-dpt. While no germinated seed was observed in the dsCYP7-treated seed wells, these wells showed a background color after staining with MTT probably due to an unknown reaction between dsRNAs and the components of the solubilization solution. Therefore, the absorbance reading was not able to produce a correct dose-response curve reflecting the genuine effect of dsCYP7 (data not shown). The germination rate of the dsCYP7-treated seeds at 8-dpt reduced significantly compared to the positive control (*p-value*< 0.05) suggesting an inhibitory effect for dsCYP7 on the broomrape germination (**Figure 1B**).

#### 3.2.2 dsCYP7 alongside negative controls

**Figure 2:**
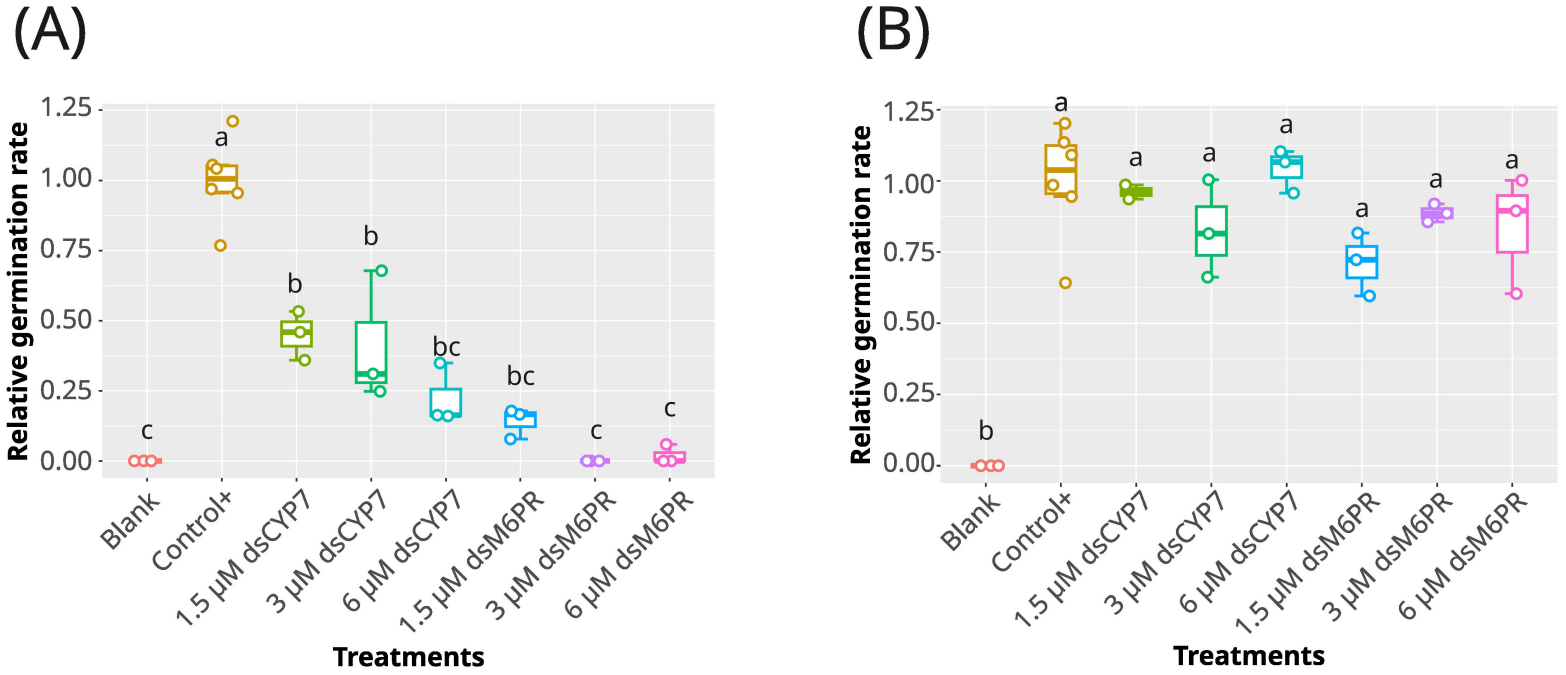
The effect of dsCYP7 and dsM6PR treatments on the germination of *P. aegyptiaca*. **(A)** The boxplot of the relative germination rates obtained in the experiment with dsCYP7 as well as dsM6PR as a non-specific dsRNA on 8-dpt. Seeds were treated with 6, 3, and 1.5 µM of each dsRNA. Seeds without +GR24 were considered as blank. **(B)** The relative germination rates of the seeds in the experiment presented in A, eight days after washing. The statistical differences among treatments were determined using Tukey's honestly significant difference test (Tukey's HSD). Treatments sharing a same lowercase letters are not significantly different at *p-value* = 0.05.

In order to examine the reproducibility of the previous experiment and to test the specificity of the dsCYP7 in inhibiting broomrape germination, another experiment was carried out by including a dsRNA targeting M6PR gene (dsM6PR, **Supplementary Figures S8A, B**) as a gene without an apparent role in the seed germination. In this experiment, three different concentrations of each dsRNAs e.g. 6, 3, and 1.5 µM were applied in the presence of 10^-6^ M (+)GR24. Unexpectedly, a reduction in seed germination was recorded in the seeds treated with both dsRNAs compared to the untreated seeds (**Figure 2A**; **Supplementary Figure S9**). In addition, the germination rates of the seeds treated with both dsRNAs were significantly lower compared to those of the positive control at 7-dpt (*p-value*< 0.05) (**Figure 2A**). The effects of dsRNAs on germination rates demonstrated a clear dose-dependent relationship, even though the differences among dsRNAs concentrations were not statistically significant (*p-value* > 0.05) (**Figure 2A**). On average, germination rates were reduced by approximately 79%, 62%, and 65% in the seeds treated with 6, 3, and 1.5 µM of dsCYP7, respectively, compared to the positive control. In addition, seeds treated with the same concentrations of dsM6PR exhibited reductions of 100%, 100%, and 85%, respectively.

To rule out that the lack of germination in the dsRNA-treated seeds was not due to the toxicity of the dsRNAs, a washing step was carried out at 8-dpt. Eight days after washing, the germination rate of the dsRNA-treated seeds recovered to the level of the positive control (*p-value* > 0.05) (**Figure 2B**) indicating that dsRNAs were not toxic. It is also worth noting that the removed dsCYP7 and dsM6PR from the wells of the seed plate during the washing step, were precipitated and purified to check whether the applied dsRNAs were degraded following seed treatment or remained intact until the end of the experiments. Agarose gel electrophoresis showed that most of the applied dsRNAs remained intact (**Supplementary Figure S10**).

**Figure 3:**
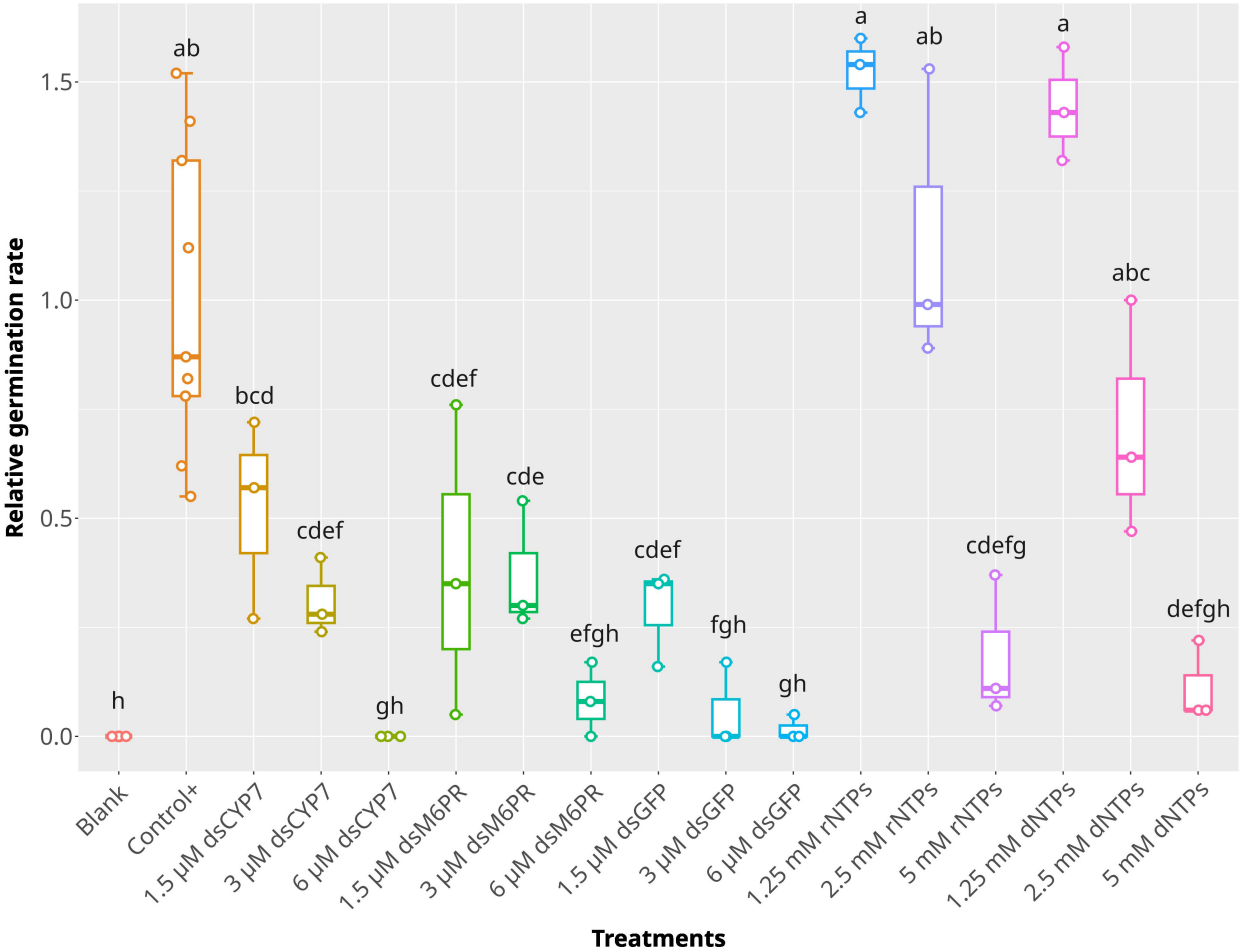
The effect of dsRNAs and nuclotides on the germination of *P. aegyptiaca*. The box plot of the relative germination rates in the experiment with dsGFP and nucleotides at 7-dpt. dsGFP was applied at 6, 3, and 1.5 µM concentration while nucleotides were applied at 5, 2.5, and 1.25 mM concentrations. The statistical differences among treatments were determined using Tukey's honestly significant difference test (Tukey's HSD). Treatments sharing a same lowercase letters are not significantly different at *p-value* = 0.05.

dsM6PR caused a greater reduction in seed germination compared to the dsCYP7. It was not clear that this reduction in the germination arose from an unknown role of the M6PR gene in the germination process or from an unknown non-specific effect of the dsRNAs on the germination. To understand it better, another experiment was conducted by including a dsRNA designed to target the green fluorescent protein (GFP) (dsGFP, **Supplementary Figures S8C, D**). Moreover, rNTPs and dNTPs at 5, 2.5, and 1.25 mM concentrations were included in the experiment as another control. Similar to the previous experiments, the germination of broomrape was considerably reduced in the wells treated with dsCYP7 and dsM6PR, with 6 µM concentration being the most effective dose (**Figure 3**). To our surprise, a similar pattern of germination inhibition was also recorded in the seeds treated with different concentrations of dsGFP, and the germination was significantly reduced compared to the positive control (*p-value*< 0.05) (**Figure 3**). Interestingly, while no germination inhibition was observed in the seeds treated with 1.25 and 2.5 mM of both types of nucleotides compared to the positive control (*p-value* > 0.05), the germination was significantly reduced in the seeds treated with 5 mM NTPs (*p-value*< 0.05), comparable to that observed in the dsRNA-treated seeds (**Figure 3**). In fact, the germination of the seeds treated with 1.25 and 2.5 mM rNTPs and 1.25 mM dNTPs increased, on average, 52%, 14%, and 44% respectively, compared to the positive control even though the differences among them were insignificant (*p-value* > 0.05). On the other hand, on average, the germination rates were reduced by 100%, 92%, 98%, 82%, and 89% in the seeds treated with 6 µM dsCYP7, dsM6PR, and dsGFP, as well as 5 mM rNTPs and dNTPs, respectively.

## 4 Discussion

Root parasitic plants belonging to the Orobanchaceae family exhibit a captivatingly intricate germination behavior. Even though it is one of the most studied aspects of the holoparasites lifecycle, our understanding of the process at the molecular level is still incomplete. Moreover, considering the dependency of the root holoparasites on their host plants which are mostly crop species, germination is a vital step, making it the focus of many studies in order to find a solution that has an edge over the chemicals to control broomrapes by preventing germination or by enhancing it!

In the current experiment, we asked the question of whether a dsRNA can be used to modulate *P. aegyptiaca* seed germination by targeting a gene involved in the germination process. The *PaCYP707A1* gene was selected as an appropriate target since it has been shown to be involved in the dormancy breaking of broomrape species (Lechat et al., 2012; Brun et al., 2019). The Initial concentration of 0.7 µM dsCYP7 was applied on the seeds and the monitoring was continued up to, usually, 7-dpt as no changes in germination are expected after this time (Pouvreau et al., 2013). Due to the lack of noticeable effect at this concentration, a more concentrated dsCYP7 of ~ 12 µM was applied on seeds as an extremum amount, which completely inhibited the seed germination. However, the application of a non-germination-specific control dsRNA targeting a gene without an apparent role in the germination process, namely dsM6PR, brought about a significant reduction in the seed germination in a dose-dependent manner, a similar pattern observed for dsCYP7. The inhibition caused by dsM6PR was confusing because it was not clear whether it was a specific effect caused by the silencing of the M6PR gene or for another reason. Hence, we included dsGFP, a non-specific dsRNA, in the experiment to obtain more insights. The similar inhibition observed following dsGFP indicated that the inhibitions caused by dsCYP7 and dsM6PR were likely non-specific effects. Moreover, an almost complete recovery of the germination in the washed dsRNA-treated seeds to a level similar to the positive control indicated that the dsRNAs were not toxic for the seeds and seeds were not killed by dsRNA treatments. This non-toxic nature of dsRNAs and the reversibility of their effects also suggest a great potential for their use as research tools in the future. It has been reported that replacing the conditioning solution containing nitrogen with a solution without it can neutralize the inhibitory effect of the nitrogen after a few days (Van Hezewijk and Verkleij, 1996). This is similar to the result obtained in the current experiment.

Since both specific and non-specific dsRNAs led to significant reductions in the seed germination of broomrape, we asked whether the lack of germination in the dsRNA-treated seeds could arise from the effect of the nucleotide bases. To address this question, rNTPs and dNTPs were included in the experiment as controls to test the effect of nitrogenous building blocks of nucleic acids. Interestingly, compared to positive control, both types of nucleotides significantly reduced seed germination at 5 mM concentration to a similar level of 6 µM dsRNAs, suggesting that the inhibitory effect of dsRNAs might arise from their nucleotide building blocks. Exogenously applied nucleotides can act as signaling molecules in plants regulating various biological processes (Witte and Herde, 2024). Some exogenously applied nucleotides have been shown to enhance root-hair growth in model plants such as *Arabidopsis* and *Medicago* (Lew and Dearnaley, 2000; Kim et al., 2006). This is in contrast with the results obtained with *P. aegyptiaca* seed germination in the current experiment.

Nitrogen deficiency has been reported to induce the production and secretion of SLs in some plant species (Yoneyama et al., 2007a, 2012). On the other hand, nitrogenous compounds and nitrogen fertilizers have been shown to reduce the germination and radical elongation in *Striga* and broomrape species (Farnia et al., 1985; Pieterse, 1991; Westwood and Foy, 1999). Similarly, nitrogen has been shown to impair the root chemotropism toward SLs and repress the formation and growth of haustoria in the hemiparasitic plant, *Phtheirospermum japonicum* (Kokla et al., 2022; Ogawa et al., 2022). These lines of reports may suggest that root parasitic plants have evolved a mechanism to perceive information on the nutritional state of their rhizosphere to leverage their germination in the finest situations fitting their heterotrophic nature.

Studies have also shown the inhibitory effect of some exogenously applied amino acids on broomrape seed germination (Vurro et al., 2006; Fernández-Aparicio et al., 2017; Arslan et al., 2023; Gibot-Leclerc et al., 2024). While this effect has been attributed to the interference with the biosynthesis of other amino acids (Vurro et al., 2006), the link between their nitrogenous moiety and their inhibitory effect worth further investigation. Furthermore, tryptone has also been reported to reduce broomrape germination in a dose-dependent manner most likely due to its _L_-tryptophan (Trp) fraction (Kuruma et al., 2021). In addition, the exogenously applied Trp is converted into indole-3-acetic acid (IAA) in *O. minor* seeds, which in turn prevents radical elongation (Tsuzuki et al., 2024). It is also worth investigating if there is any link between nucleotides and the inhibitory effect of IAA in the seeds of root parasites.

Modulating the broomrape seed germination has been achieved via the application of miPEPs by regulating their corresponding *MIR* genes, and subsequently, post-transcriptional gene silencing of their corresponding target genes, leading to germination inhibition when applied exogenously on the seeds at appropriate amounts (Tourneur et al., 2024). Therefore, the process of germination seemed accessible to be modulated through RNA silencing via the exogenous application of molecules such as dsRNAs. In our study, however, dsRNAs impacted the germination in a non-specific manner indicating that their application for gene silencing in the germination stage will require some refinements. One suggestion would be to analyze the levels of the target gene transcripts after treatment with dsRNAs that could provide a clue as to whether the exogenous RNAi is effective, at least, at the transcript level or not. Nonetheless, as it seemed that the non-specific observed inhibitory effects of dsRNAs on the seed germination originated from nitrogenous nucleotides thereof, the application of exogenous RNAi at the germination level may confront a challenge, implying on the importance of finding solutions to minimize the non-specific effects of dsRNAs.

## Data Availability

The raw data supporting the conclusions of this article will be made available by the authors, without undue reservation.
